# A microcomputer-based injection system for investigating the influence of atmospheric pressure on chromatographic response in the analysis of gases

**DOI:** 10.1155/S1463924683000334

**Published:** 1983

**Authors:** Brian R. Wybrow, E. J. Greenhow

**Affiliations:** 1 British Gas Corporation: Research and Development Division London Research Station Michael Road London SW6 2AD UK; 2 Department of Chemistry Chelsea College Manresa Road London SW3 6LX UK


Journal of Automatic Chemistry, Volume 5, Number 3 (July–September 1983), pages 124–135

### A microcomputer-based injection system for investigating the influence of atmospheric pressure on chromatographic response in the analysis of gases

**Brian R. Wybrow**

*British Gas Corporation: Research and Development Division, London Research Station, Michael Road, London SW6 2AD*

**and E. J. Greenhow**

*Department of Chemistry, Chelsea College, Manresa Road, London SW3 6LX*

### Introduction

In gas chromatographic analysis, in order to assist automation and to improve precision, gas samples are usually injected by means of a gas-sampling valve which is connected to a sample loop of fixed volume. Sample gas is passed through the loop for a time sufficient to ensure that the loop contains only the sample under investigation. The sample gas supply is then shut off to allow the gas pressure in the loop to equilibrate to atmospheric pressure. Injection then follows. This procedure constitutes the standard 'stopped-flow' technique of injection. The column effluent passes directly from the detector into the atmosphere, so as long as no atmospheric pressure change takes place during the analysis, the 'injected' and 'detected' sample are exposed to the same atmospheric pressure. Through the operation of Boyle's law, an increase in atmospheric pressure will cause an increase in the mass of sample injected, but the concentration of sample in the detector cavity will tend to remain constant because of its dilution by carrier gas molecules whose number, predicted from the operation of Boyle's law, will vary linearly with atmospheric pressure [1 and 2]. Variation in atmospheric pressure is therefore predicted to have a minimal influence on response for a concentration-sensitive detector, but it should give rise to significant changes in response for a mass-sensitive detector [2]. Boček *et al.* [3], have shown that for a flame ionization detector, a type of mass-sensitive detector, the situation is further complicated by the effect of atmospheric pressure on ionization efficiency.

For these reasons, and because of the additional need to analyse samples of gas supplied at subatmospheric pressure, an investigation into injection technique was initiated. The aim was to improve both accuracy and precision when these detectors are used. Favourable experiences with an already proven microcomputer-based sequence controller, which uses specially written software and hardware built to control an automatic gas chromatograph, led to its adoption for the investigation. The chromatograph utilizes the separation techniques developed by Deans *et al.* [4], and is normally used for isothermal analysis of $H_2$, $O_2$, $N_2$, CO, $CH_4$, $CO_2$, $C_2H_4$ and $C_2H_6$. The current work on injection technique has concerned only nitrogen. The sequencer operates four pneumatic valves, which are clamped together, and which, with associated needle valves, form an 'Injection Module'. This controls the passage of sample gas into the sample loop of the gas chromatograph, isolation of the loop from the laboratory atmosphere, and its evacuation by a vacuum pump, which is also under microcomputer control. A combination of an absolute pressure transducer with a specially constructed digital barometer, which contains electronic comparator circuits, supplies the microcomputer with signals that satisfy conditional tests (called 'Interrupts') created for its input ports within sequential control programs written in a high-level language. Any desired pressure in the loop can thus be achieved by placing the conditional tests before, or after, steps in the sequential control program which involve valve operation, and by setting appropriate values on the barometer.

This paper describes the functional parts of the injection system, explains how it is used, and evaluates the results obtained for various applications. The results are discussed, and predictions are made about the influence of variations in atmospheric pressure on chromatographic response when analysing gases and liquids, using concentration- and mass-sensitive detectors. Conclusions are then drawn and recommendations are made.

### Apparatus

### *The microcomputer system*

The microcomputer system is called PROSE (standing for PROgrammable Sequencing Equipment) and is based on a Zilog Program Development Station, which comprises a microcomputer (using the Zilog Z80 microprocessor), a floppy disc unit and a terminal. A special software package ('Interpreter'—written by members of the Projects Group, Computer Services Department, Economic Planning Division, British Gas Corporation), loaded from disc, enables the user to write control

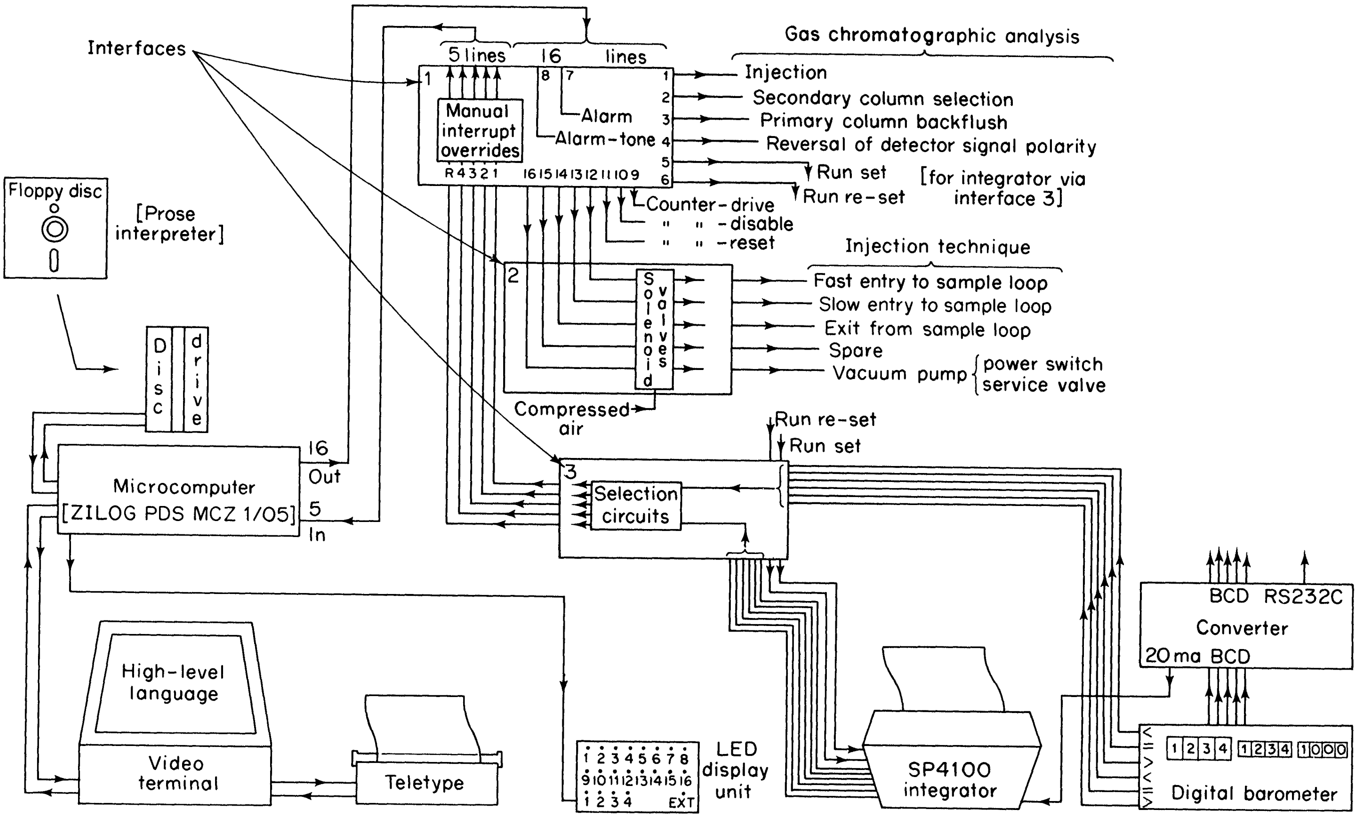

*Figure 1. Functions of control lines of system.*

programs in an easily assimilated, high-level language, also called PROSE. Three specially constructed electronic interfaces enable the microcomputer to drive and receive signals from external equipment.

The functions of the 16 output and five input control lines are shown in figure 1 and summarized in table 1. Figure 2 shows how the sample-handling and pressure-measuring equipment is used in the complete chromatographic system. Output lines can be activated at absolute or relative times and sequences can be repeated either continuously or a pre-defined number of times. Alternatively, control lines can be activated immediately by direct software commands from the terminal.

Sequences can be saved on disc, and can be rapidly checked and edited before execution on the controlled equipment. Or, if desired, checking can be carried out in real-time by switching off Interface 1 and observing an LED display (see figure 1), which reveals the state of each of the 16 output control lines and each of the four, general input lines. An 'external wait' signal on the LED display indicates that the microcomputer is waiting for an Interrupt to be satisfied on any of the five input lines; the LED is extinguished when the Interrupt is satisfied.

During sequence execution a status report can be obtained by pressing the 'R' key on the terminal. Sequence execution can be terminated at any time by pressing the 'CONTROL' and 'S' keys simultaneously; absolute time and current position in the sequence will then be reported. A further aid to using the system is the ability to place descriptive comments in brackets after each line action command.

The five input lines allow the user to interrupt program execution by placing software Interrupts at selected points in the

*Table 1. PROSE control lines.*

| | Output | |
|---|---|---|
| | Number | Function |
| Control jobs | 1 | Injection valve |
| | 2 | Secondary column selection |
| | 3 | Primary column backflush |
| | 4 | Reversal of detector signal polarity |
| | 5 | Set integrator to run condition |
| | 6 | Reset integrator to standby condition |
| Inform user | 7 | Operate alarm |
| | 8 | Select alarm tone |
| | 9 | Digital readout counter drive |
| | 10 | Counter disable |
| | 11 | Counter reset |
| Injection technique | 12 | Fast entry to sample loop |
| | 13 | Slow entry to sample loop |
| | 14 | Exit from sample loop |
| | 15 | Spare |
| | 16 | Vacuum pump { power switch / service valve |

| Input | |
|---|---|
| Number | Function |
| 1 | Software interrupt |
| 2 | Software interrupt |
| 3 | Software interrupt |
| 4 | Software interrupt |
| Remote start | Software interrupt |

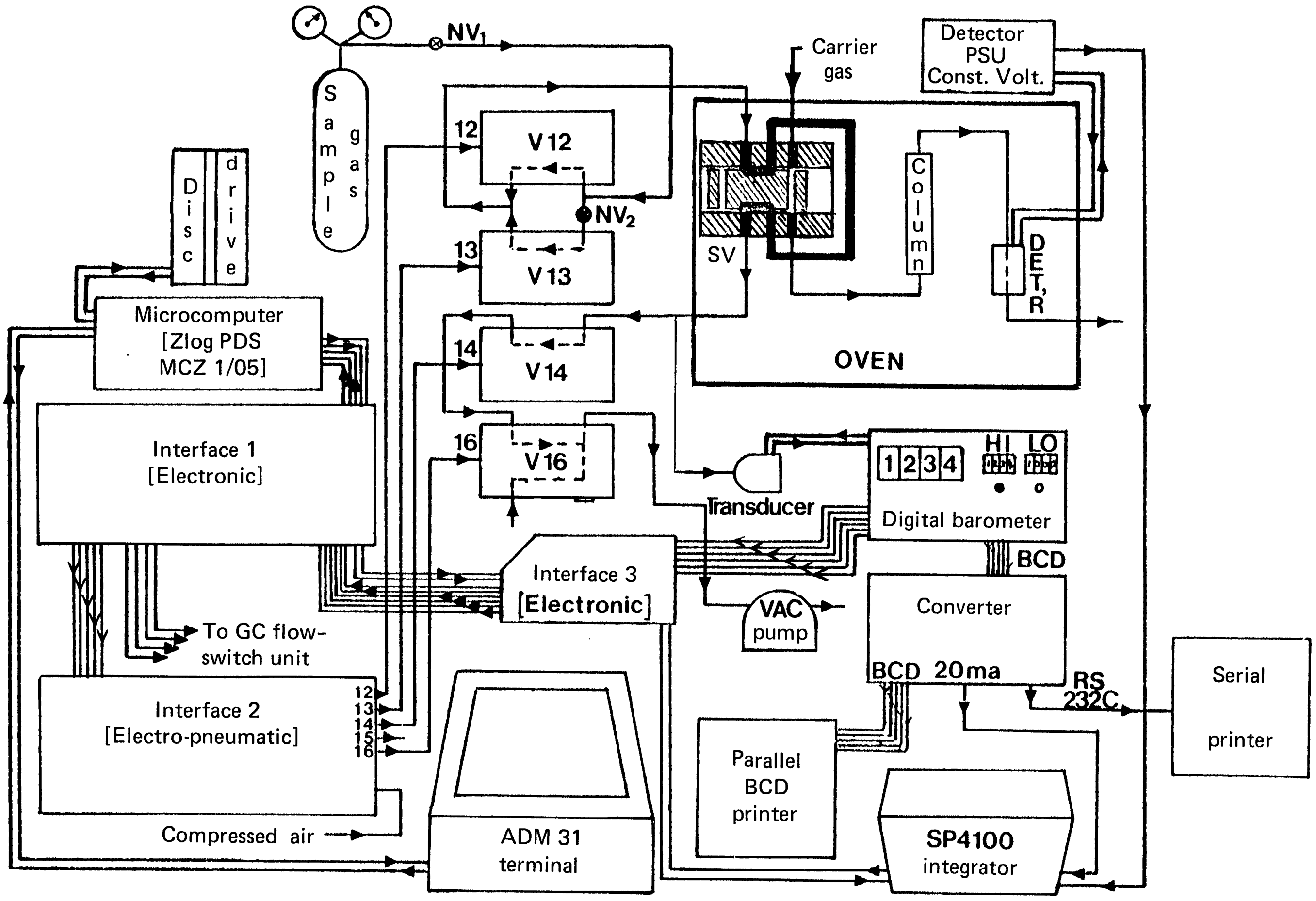

*Figure 2. Apparatus for microcomputer control of sample injection technique and chromatographic analysis. Where V12 to V14, and V16 = pneumatic valves; SV = pneumatically activated gas sampling valve; PSU = power-supply unit; BCD = parallel binary coded decimal standard, for communicating with printer; 20 ma = serial current loop standard, for printing pressure, using SP4100 integrator; and RS232C = serial, voltage level communication standard.*

sequential control program. Then, only when the Interrupts are satisfied by signals from external measuring equipment, for example from the digital barometer or the integrator handling the chromatographic data or a simple switch (see figure 3), will the next step in the program be executed. Thus, for example, the command 'LWAIT 1' (abbreviated LW 1), will suspend execution of a sequence until Input line 1 is at a logical 'low' level. A simple, yet very useful, application of Interrupts allows either continuous or single analyses to be performed at the user's discretion by placing the Interrupt 'LW 4' before the 'REPEAT' command in a sequence. A manual override switch, SW4, in Interface 1 (which is connected to Input line 4) being placed in either the 'down for low-level', 'pass' position, or the 'up for high-level', 'stop' position respectively.

### *Interfacing equipment*

There are three interfaces connecting the microcomputer to the various functional parts of the system (see figures 1 and 2).

Interface 1 enables Output line 1 to operate a six-port pneumatic injection valve situated inside the chromatographic oven and enables Output lines 2 and 3 to operate other valves concerned with the gas chromatographic analysis, all via a switching unit containing solenoid valves; it also buffers the microcomputer from all other equipment. A digital counter (with a four-digit seven-segment display), which is driven via the Interface by Output lines 9, 10 and 11, and a two-tone alarm inside the Interface driven by Output lines 7 and 8, can be used to inform the operator when certain stages in the analysis have

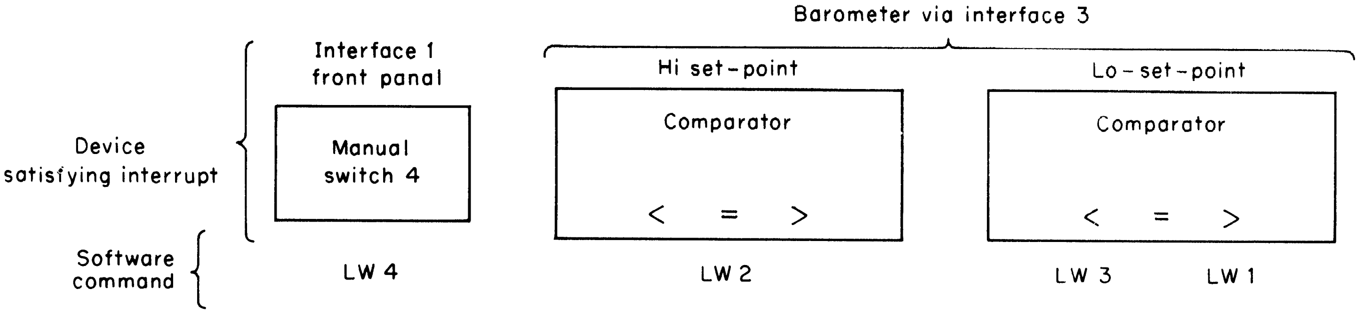

*Figure 3. Selections made for interrupts.*

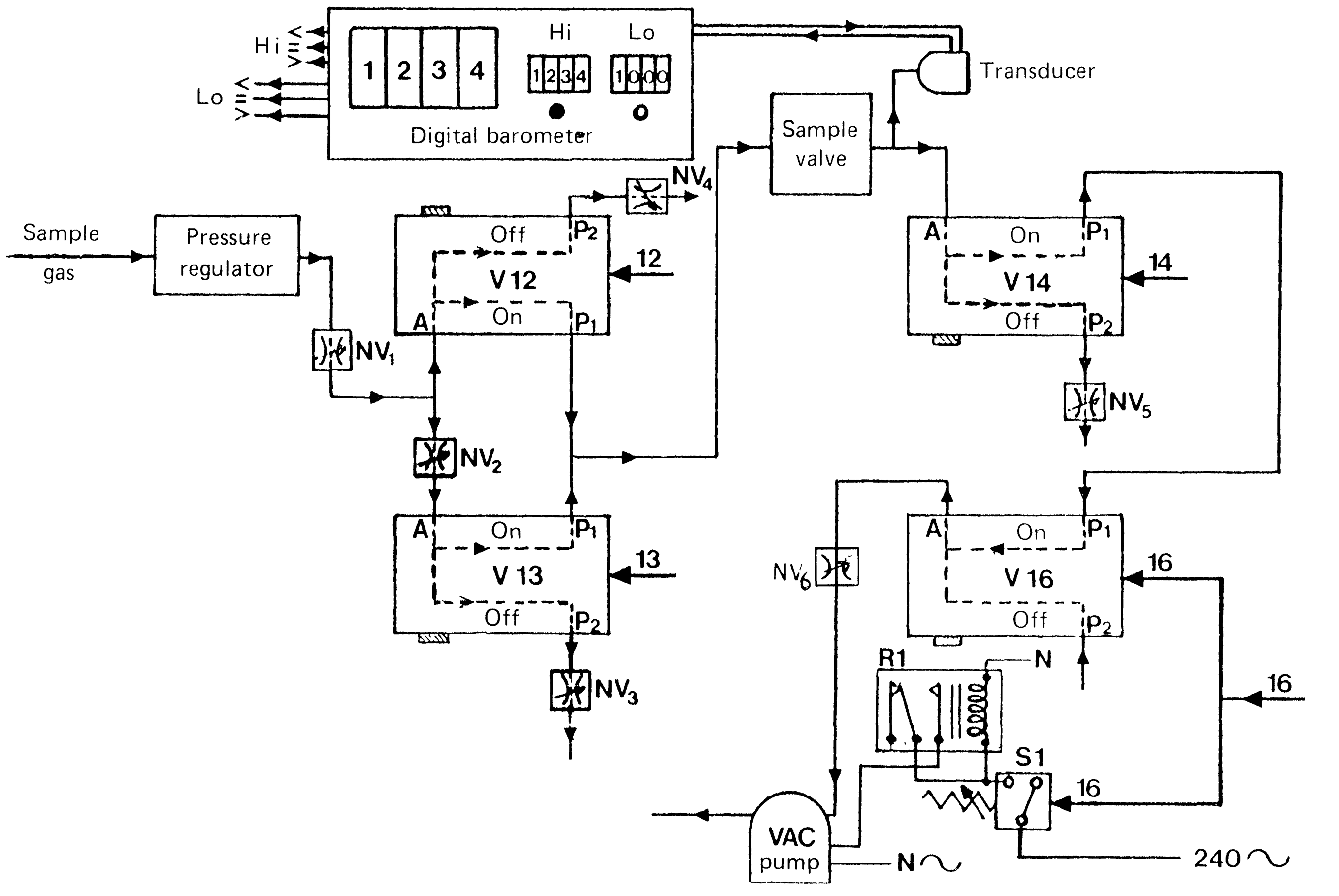

*Figure 4. Apparatus for automatic control of sample injection pressure. Where NV1 = coarse control needle valve; NV2, NV3, NV4, NV5 = fine control needle valves; A, P1, P2 = valve ports; ←12 = PROSE control line 12; S1 = pneumatic switch; and R1 = heavy duty relay.*

been reached. These devices are under software control, so their operation can be linked with that of any of the other equipment driven by the microcomputer. A 'REMOTE START' button (RS1), linked to Input port 5 enables analyses to be initiated from the Interface, or, more conveniently, from wherever the button may be placed. Four switches, SW1 to SW4, located on the front panel of the Interface provide logical 'high' or logical 'low' level signals to Input ports 1 to 4; red/green LEDs indicate the logic level chosen on each switch. These switches can serve as Interrupt controllers when used in conjunction with appropriate Interrupts created in PROSE software.

Interface 2 is an electro-pneumatic interface which, under the control of the microcomputer via Interface 1, drives the vacuum pump and the four pneumatic valves in the Injection Module shown in figure 4. The interface provides means for manual or automatic admission of gas to the sample loop of the chromatograph and for isolating the gas within the loop, at a pre-set pressure, ready for injection. It was designed to allow any device providing TTL-compatible, latched signals to switch compressed air to five independent outputs.

The main functional element in the Interface is the Solenoid control board 5 (see circuit diagram in figure 5). This takes the five PROSE outputs 12 to 16 from output socket 02 of Interface 1 to the DIL reed relays RY7 to RY11 on the board. These provide +24V to the five solenoid valves inside the pneumatic control module (see figure 6), which drive the pneumatic valves 12 to 16 in the Injection Module. Manual override switches, SW11 to SW15 (figure 5), allow manual operation of the pneumatic valves 12 to 16 independently of the microcomputer—thereby assisting program evaluation and general setting-up of the system.

Interface 3 is an opto-isolated, manual switching interface, which allows the operator to select the source of hardware (the digital barometer or the SP4100 integrator) which, after further selection via the manual override switches inside Interface 1, will satisfy Interrupts created in sequential control programs. Figure 3 summarizes selections made for four of the input lines when using the digital barometer.

When automatically optimizing column switching and back-flushing times in the analysis, any one of the six external time function outputs from the integrator is linked via Interfaces 3 and 1 to a chosen input port. Interface 3 also allows the microcomputer to supply, via Interface 1, 'RUN SET' and 'RUN RE-SET' signals for starting and stopping integration of the detector signal by the integrator.

### *Optimization of column switching and back-flushing times*

The automatic gas chromatograph incorporates three chromatographic columns (one primary and two secondary) in a column switching and back-flushing routine which uses 'external to the column oven' flow-switching techniques, after Deans *et al.* [4]. The times of secondary column selection or primary column back-flushing respectively are automatically optimized. A ROM Overlay BASIC program in the SP4100 integrator, together with a time-incrementing or time-decrementing PROSE program in the microcomputer, are used

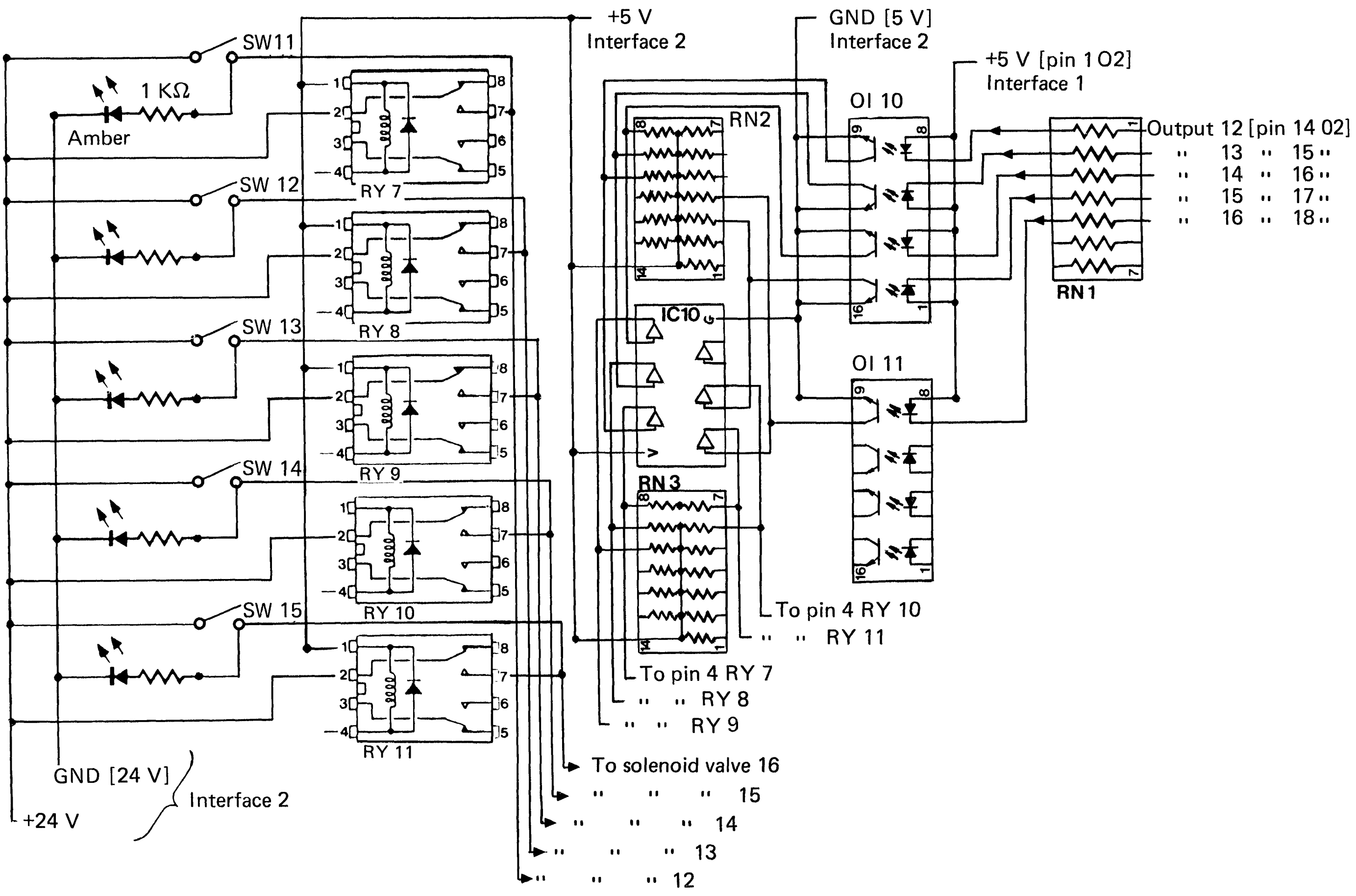

*Figure 5. Circuit diagram of solenoid control board 5, in Interface 2.*

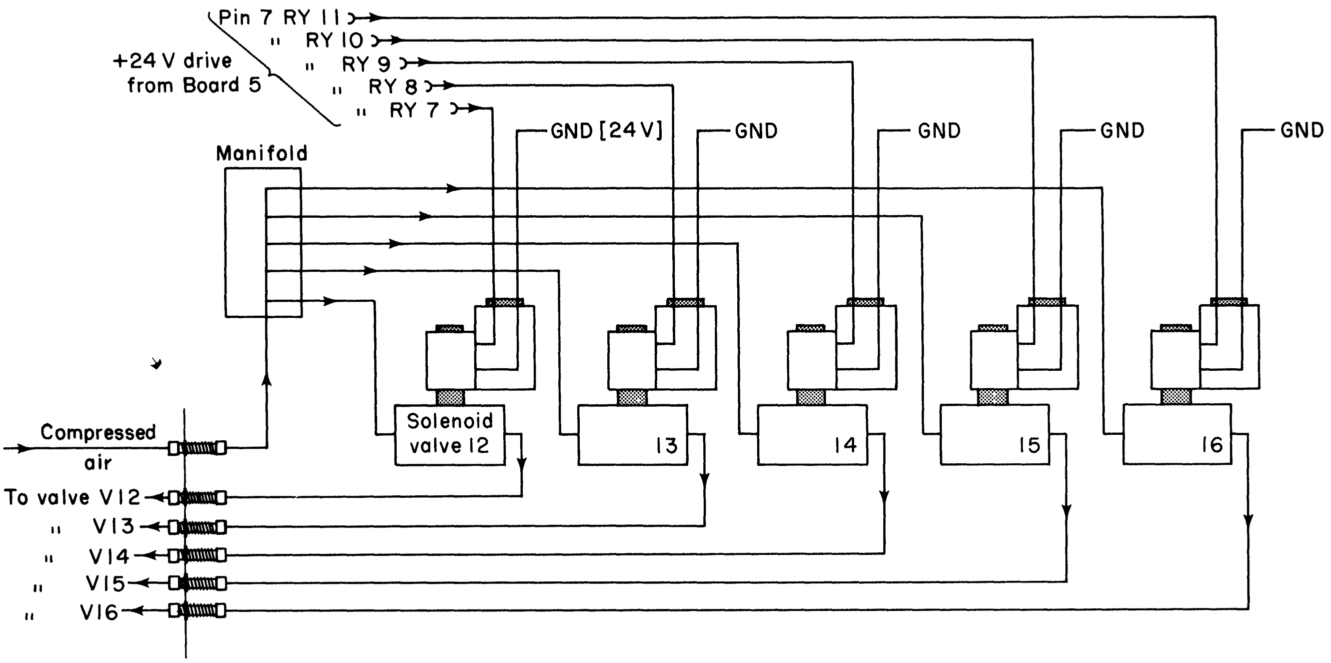

*Figure 6. Pneumatic control module in Interface 2.*

for this. The PROSE program repeatedly changes the times of column switching and column back-flushing, to a discrimination of 0·1 s, provided that an Interrupt created for one of the input lines is satisfied by a signal from one of the external time function outputs of the integrator. The voltage level on this output can be made to be dependent on the outcome of a conditional test within the BASIC program in the integrator which looks at the areas of strategic peaks in the chromatographic analysis. So the integrator can be used to 'inform' PROSE when it should cease changing the relevant switching time. This technique automatically provides the minimum time at which to initiate primary column back-flushing, and the range of times within which to make secondary column selection.

### Control of injection technique

### *Outline*

The use of automatic techniques to reach a desired pressure of gas in the sample loop of a chromatograph requires special equipment, particularly for attaining subatmospheric pressures. In this investigation the use of a conventional motorized pressure regulator has been avoided. Instead a pressure transducer and specially constructed digital barometer were employed, together with software in the microcomputer, to open and close the valves in an Injection Module, which controls admission and exit of sample gas to and from the sample loop. In order to deal safely with flammable gases these valves are pneumatic and are driven remotely by means of low-power solenoid valves contained in Interface 2. This ensures isolation of the sample gas from potential sources of electrical discharge.

In order to determine the mechanism and the extent to which variations in atmospheric pressure can alter responses in gas chromatographic analysis, an absolute pressure transducer, referred to vacuum, is used to isolate the sample loop, and hence the mass of sample injected, from the influence of atmospheric pressure. By setting appropriate values on the digital barometer, injections can be made at any desired pressure of sample gas in the loop; then variations in atmospheric pressure are only able to influence the contents of the detector cavity. Alternatively, a 'stopped-flow' technique of injection can be simulated by setting the digital barometer to the prevailing atmospheric pressure before each injection.

### *Pressure measurement*

The digital barometer (see figures 1, 2 and 4) contains two electronic comparator circuits; these compare the absolute pressure of gas in the sample loop with 'LO' and 'HI' values set by the user on each of two Binary Coded Decimal (BCD) thumbwheel switches on the front panel of the barometer. The voltage level on any of three possible outputs (reading $\langle, =$ or $\rangle$, set-point) from each comparator circuit can be tested by software Interrupts placed by the user at strategic points in the analytical sequential control program. These Interrupts can be preceded, or followed immediately, by operation of any of the sample gas flow control valves 12, 13, 14 and 16, which are driven by PROSE Output lines having the corresponding number (see figures 2 and 4).

### *Sample introduction*

Sample gas enters the injection system (shown in figure 4) via coarse control needle valve $NV_1$, while valve 12 controls fast admission of gas to the loop. Valve 13, preceded by fine control needle valve $NV_2$, controls slow admission of gas to the loop, and valve 14 controls exit of gas from the loop. Valve 16 is concerned with use of the vacuum pump for attaining subatmospheric pressures; the pump is switched on by PROSE Output line 16 via pneumatic switch S1 and relay R1. Valve 16 ensures that the vacuum pump is always vented to atmosphere when switched off—thus preventing oil from being drawn into the vacuum service line.

Needle valve $NV_3$ eliminates pressure surges by preventing build-up of pressure behind valves 12 and 13 when they are closed. $NV_4$ allows the dead space between ports A and $P_2$ in valve 12 to be purged with sample gas; in normal use $NV_4$ is shut. $NV_5$ enables atmospheric pressure to be monitored when valve 16 is connected to valve 14 for attaining subatmospheric pressures. When valve 16 is not connected, port P1 on valve 14 is open to atmosphere and $NV_5$ is permanently closed; isolation of the loop from the laboratory atmosphere is then achieved by turning valve 14 off. $NV_6$ controls the rate of evacuation of the sample loop and thus ensures that the 'LO' set-point of the digital barometer is not passed too rapidly when preparing for injection at subatmospheric pressure. For convenience, the regulator controlling the pressure of gas leaving the cylinder of sample gas is set to deliver gas at greater than the maximum pressure required in the investigation. However, care must be taken to ensure that the proof pressure of the transducer, and the maximum working pressure of the gas sampling valve, are not exceeded.

### Using the injection system

### *Outline*

Three PROSE programs, 'ATMOS', 'SUPERATMOS', and 'SUBATMOS', have been written for injection with the aid of the digital barometer at ambient, super- and subatmospheric pressures respectively. Two other programs, 'AGCSTOP' and 'AGCPURGE', have been used to investigate injection technique without the aid of the digital barometer. AGCSTOP simulates a situation in which no automatic feedback facilities are available and where only entry of sample gas to the loop, and operation of the injection valve, are under automatic control. AGPURGE simulates a situation in which only the injection valve is under automatic control. These are two situations which typify injection technique in the analysis of gases.

In all cases the program relating to implementation of injection technique precedes that employed for the chromatographic analysis.

Operation of the system is exemplified by the hypothetical program 'TEST' shown in figure 7, which simulates a simple application and demonstrates the principles of the software

```
LI TEST

TEST
  1> ON 14 (OPEN LOOP EXIT VALVE)
  2> WA 3 (WAIT THREE SECONDS)
  3> ON 12 (OPEN FAST ENTRY VALVE)
  4> WA 10 (WAIT TEN SECONDS)
  5> OFF 14 (CLOSE LOOP EXIT VALVE)
  6> LW 1 (INTERRUPT)
  7> OFF 12 (CLOSE FAST ENTRY VALVE)
  8> WA 3 (WAIT THREE SECONDS)
  9> ON 13 (OPEN SLOW ENTRY VALVE)
 10> LW 2 (INTERRUPT)
 11> OFF 13 (CLOSE SLOW ENTRY VALVE)
 12> PERFORM ANALYSIS (EXECUTE ANALYSIS PROGRAM)
 13> END
```

*Figure 7. Full listing of PROSE program 'TEST'.*

```
FL SUPERATMOS

SUPERATMOS
  1> ON 14 (OPEN LOOP EXIT VALVE–MONITOR ATMOSPHERIC PRESSURE)
  2> LW 4 (INTERRUPT ON INPUT LINE 4–SATISFIED BY MANUAL SWITCH)
  3> WA 3 (WAIT 3 SECONDS)
  4> ON 12 (OPEN VALVE PROVIDING FAST ENTRY OF SAMPLE GAS INTO LOOP)
  5> LW 4 (MANUAL INTERRUPT)
  6> OFF 14 (CLOSE LOOP EXIT VALVE)
  7> LW 1 (TEST FOR PRESSURE> SET-POINT ON LO SET-POINT COMPARATOR)
  8> OFF 12 (PREVENT ENTRY OF SAMPLE GAS INTO LOOP)
  9> WA T9 (WAIT T9 SECONDS–RECOMMEND T9=2 SECONDS)
 10> PER AGCX1 (EXECUTE SUB-SEQUENCE AGCX1)
 11> END

AGCX1
  1> ON 13 (OPEN VALVE PROVIDING SLOW ENTRY OF GAS INTO LOOP)
  2> LW 2 (TEST FOR PRESSURE=SET-POINT ON HI SET-POINT COMPARATOR)
  3> OFF 13 (STOP SLOW ENTRY OF GAS INTO LOOP)
  4> PER AGCX2 (EXECUTE SUB-SEQUENCE AGCX2 [THIS PERFORMS THE ANALYSIS])
  5> LW 4 (MANUAL INTERRUPT)
  6> ON 14 (OPEN LOOP EXIT VALVE–MONITOR ATMOSPHERIC PRESSURE)
  7> WA 5 (WAIT 5 SECONDS)
  8> LW 4 (MANUAL INTERRUPT)
  9> ON 12 (OPEN VALVE PROVIDING FAST ENTRY OF SAMPLE GAS INTO LOOP)
 10> WA 30 (WAIT 30 SECONDS I.E. PURGE LOOP AT FAST RATE FOR 30 SECONDS)
 11> LW 4 (MANUAL INTERRUPT–HOLD PURGE BEYOND 30 SECONDS IF DESIRED)
 12> OFF 14 (CLOSE LOOP EXIT VALVE–ALLOW PRESSURE IN SAMPLE LOOP TO BUILD UP)
 13> LW 1 (TEST FOR PRESSURE > SET-POINT ON LO SET-POINT COMPARATOR)
 14> OFF 12 (PREVENT FURTHER ENTRY OF SAMPLE GAS INTO LOOP)
 15> WA 2 (WAIT 2 SECONDS)
 16> RE N6 (REPEAT SEQUENCE AGCX1 N6 TIMES)
 17> END

AGCX2
  1> ON 5 (SET INTEGRATOR TO RUN CONDITION)
  2> ON N9 (INJECT SAMPLE—AUTO INJECTION, N9=1;—MANUAL INJECTION, N9=0)
  3> WH 2 OFF 5 (DE-ACTIVATE RUN-SET SWITCH,—INTEGRATOR STAYS IN RUN MODE; RUN)
  4>            (RE-SET IS AVAILABLE FROM INTEGRATOR)
  5> WH 150 ON 2 (SWITCH OUTLET OF PRIMARY COLUMN TO SECONDARY COLUMN 2)
  6> WH T1 ON 4 (REVERSE SIGNAL POLARITY; T1=276)
  7> WH 420 OFF 3 (DE-EN BACKFLUSH VALVE, I.E. EXECUTE BACKFLUSH OF PRIMARY COL)
  8> WH 430 OFF N9 (RETURN INJECTION VALVE TO FILL POSITION)
  9> WH T3 ON 14 PU 7 (LOOP EXIT VALVE NOW OPEN; T3=432; PULSE ALARM)
 10>                  (MONITOR ATMOSPHERIC PRESSURE)
 11> WH T4 ON 12 (LOOP NOW BEING FAST PURGED WITH SAMPLE; T4=440)
 12> PU 7 (PULSE ALARM)
 13> WH T5 OFF 14 (CLOSE LOOP EXIT VALVE; T5=540)
 14> (PRESSURE NOW BUILDING UP TO FIRST SET-POINT)
 15> LW 1 (TEST FOR PRESSURE > SET-POINT ON LO SET-POINT COMPARATOR)
 16> OFF 12 (CLOSE VALVE PROVIDING FAST ENTRY OF GAS INTO LOOP)
 17> WH 826 OFF 2 (SWITCH OUTLET OF PRIMARY COLUMN TO SECONDARY COLUMN 1)
 18> WH 827 OFF 4 (RETURN POLARITY TO STANDBY STATE)
 19> WH 846 ON 3 (RETURN BACKFLUSH VALVE TO STANDBY STATE)
 20> (I.E. ENERGISED=FORWARD FLOW)
 21> WH 1020 PU 7 ON 6 (PULSE ALARM AND RE-SET INTEGRATOR)
 22> WH 1022 OFF 6 (ALLOW INTEGRATOR TO BE SET NEXT TIME)
 23> WH 1080 PU 7 END (PULSE ALARM AND END SEQUENCE)
```

*Figure 8. Full listing of PROSE program 'SUPERATMOS'.*

used in PROSE application programs, one of which (SUPERATMOS), is shown in figure 8.

Before using the system for attaining a particular injection pressure, the openings of needle valves, $NV_1$, $NV_2$, and $NV_3$ (see figure 4), have to be set to satisfy the demands made by the chosen 'LO' and 'HI' set-points of the digital barometer on the supply of sample gas. Alternatively, if desired, the needle valves can be left alone and the regulator controlling the supply of sample gas can be adjusted instead.

*Explanation of program 'TEST' (shown in figure 7)*

*Note:* TEST is for use without valve 16; needle valve $NV_5$, connected to valve 14, is therefore permanently closed.

Step 1, 'ON 14', turns on valve 14 and allows the atmospheric pressure in the laboratory to be monitored.

Step 3, 3 s later, turns on valve 12 so that the sample loop is now being rapidly purged with sample gas.

Step 5, 10 s later, turns off valve 14 so that pressure starts to build up in the loop. However, step 6 immediately tests whether or not the pressure is greater than that on the 'LO set-point' comparator of the digital barometer. As soon as it *is* greater, Interrupt 'LW 1' is satisfied at step 6, and step 7 is implemented; this turns off valve 12 and hence the supply of sample gas to the loop. The rising gas pressure has thus been caught at some value just greater than that set on the 'LO set-point' comparator.

Three seconds later, step 9 turns on valve 13 and hence supplies gas to the loop at a rate chosen to be low enough to ensure that when the pressure set on the 'HI set-point' comparator is reached, Interrupt 'LW 2' at step 10 is implemented and valve 13 is switched off in step 11 at nearly the same pressure. The gas is now in the loop at the required 'set-point' pressure and

PROSE executes step 12 which 'PERFORMS' the program 'ANALYSIS'.

$NV_3$ on outlet $P_2$ of valve 13 ensures that pressure does not build up behind valve 13 when it is off. Without $NV_3$, this build-up of pressure would result in a sudden surge of gas into the loop when valve 13 was opened and would cause the 'HI' set-point to be exceeded; this is particularly important for repeat analyses and inter-run 'fills'. Interrupt 'LW 2' at step 10 in TEST would then either not be implemented, or if it was, the following step 'OFF 13' would be implemented only when the pressure had already exceeded the 'HI' set-point. This again emphasizes the importance of matching the needle valve settings to the chosen set-points. The 'LO' set-point needs to be close enough to the 'HI' set-point to ensure that the slow 'fill' does not take too long, but it must not be so close as to result in the 'HI' set-point being exceeded during the fast 'fill'.

### *Other programs*

In order to inject gas at the prevailing atmospheric pressure, this pressure is first measured, and the 'HI' set-point comparator is set to the measured value. ATMOS is used to effect automatic injection at atmospheric pressure. Alternatively, AGCSTOP can be used without having to monitor atmospheric pressure. Either method simulates a 'stopped-flow' technique of injection.

SUBATMOS is used for attaining subatmospheric pressures with the vacuum pump.

### *Use of a printer*

The incorporation of a printer greatly simplifies application of the injection technique since it is then only necessary to aim roughly for the desired injection pressure using the fast 'fill'; the print-out, which can be generated as often as desired, then confirms the pressure, just before injection, at injection and then during the analysis. A device eminently suited to this role is the SP4100 integrator. This has been interfaced to the digital barometer via a Converter unit (shown in figures 1 and 2) which converts pressure data from the barometer, in parallel BCD form, to serial 20 mA current loop form for use by the integrator. Pressure data can conveniently be stored in an array within 'BASIC' program in the integrator; this aids further computation of results.

Alternatively, an auxiliary, serial, or parallel BCD, printer can be used via the Converter unit.

### Results

### *Using the digital barometer*

For an ideal gas, Boyle's law predicts that, at constant temperature, the number of moles of gas injected will be directly proportional to the pressure of gas in the sample loop. Thus, for a thermal conductivity detector, the peak area response, which is directly proportional to the concentration of sample in the detector cavity and hence also to the number of moles injected, will be proportional to this pressure. This is confirmed by the regression data, shown in table 2, for nitrogen injected at a constant atmospheric pressure of 1008 mbar, over the pressure range vacuum to 1690 mbar absolute. So for injections made always at the prevailing atmospheric pressure, these results might at first suggest that peak area response should increase linearly in this way with atmospheric pressure. However, for a thermal conductivity detector, the detector cavity is subject to the compensatory diluting effect [1 and 2] of increasing atmospheric pressure on sample concentration, through the increase in the number of moles of carrier gas present. The net result will therefore be from a combination of these two effects acting in opposition and is shown graphically, for nitrogen, as the lowest plot in figure 9.

The influence of atmospheric pressure on peak area response, through its effect on the contents of the detector cavity alone, has been quantified by carrying out injections with the digital barometer set for absolute pressures of 1080, 1180, 1390 and 1690 mbar. This has produced the other plots shown in figure 9. Linear regression data for all these plots are given in table 2.

### *Without the barometer*

AGCSTOP and AGCPURGE demonstrate the advantages of a 'rapid-purge', 'stopped-flow' technique of injection over a 'slow-purge' technique involving injection whilst purging the loop with sample gas. The levels of precision obtained with each technique for six analyses of nitrogen in each case were examined. AGCSTOP gave a 99% confidence interval of ±0·06% relative about the mean peak area, whilst the same interval with AGCPURGE was ±0·97%.

The lower precision obtained from AGCPURGE is due to incomplete flushing out of carrier gas from the sample loop during the limited time available betweeen successive injections.

*Table 2. Regression data for plots of peak area V atmosphneric pressure shown in figure 9 and for injections at constant atmospheric pressure.*

| Injection pressure (mbar) | Number of results (N) | Degrees of freedom (N−2) | Significance level (%) | Tabulated value of correlation coefficient ($r$) | Calculated value of correlation coefficient ($r$) | Slope ($m$) | $y$ intercept (c) | Range in time (days) |
|---|---|---|---|---|---|---|---|---|
| | | | | | (For regression of $y$ on $x$) | | | |
| Atmospheric | 14 | 12<br>25† | 0·1 | ±0·780<br>±0·597† | +0·794 | +2332·4 | 6055450 | 60 |
| 1080 | 31 | 29<br>30 | 0·1 | <br>±0·554† | −0·833 | −7016·3 | 16069068 | 61 |
| 1180 | 8 | 6 | 1·0 | ±0·834 | −0·911 | −6781·6 | 16637014 | 54 |
| 1390 | 5 | 3 | 2 | ±0·934 | −0·954 | −11403 | 23023827 | 44 |
| 1690 | 5 | 3 | 5 | ±0·878 | −0·910 | −7201·4 | 21178250 | 18 |
| 0 to 1690 (at a constant atmospheric pressure of 1008) | 8 | 6 | 0·1 | ±0·925 | +0·99997 | +8248·4 | 48132 | 0 |

† Nearest tabulated value.

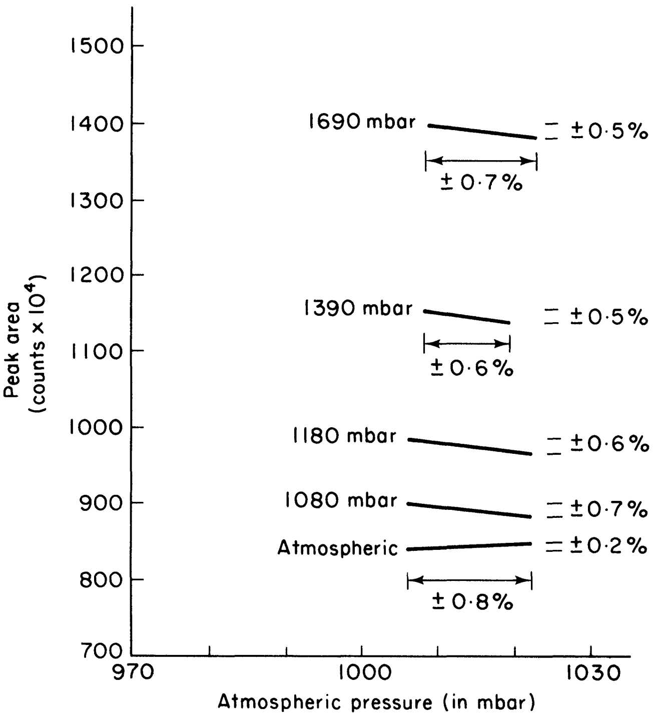

*Figure 9. Peak area response (counts × $10^4$) versus atmospheric pressure (mbar) for injection of nitrogen: (a) always at the prevailing atmospheric pressure (bottom curve) and (b) at absolute pressures of 1080, 1180, 1390 and 1690 mbar (other curves).*

This could happen if the chromatographer attempted to maintain precision without measuring flow rate. The sample purge rate in this case would be kept at some very low value, near zero, in order to both conserve sample and avoid over-pressurization of sample gas in the loop. A higher purge rate would provide better purging, but it would require either precise measurement or precise control and could give rise to over-pressurization. Also, when the sampling valve is situated *inside* the chromatographic oven, variable flow rates will give rise to variable residence times of sample gas in the loop and this will alter response because the density of the gas sample will vary.

### Discussion

When a sample of gas is injected at a constant absolute pressure, and when a concentration-sensitive detector is employed, there should be a rectangular hyperbolic relationship between response and atmospheric pressure if dilution of the eluted sample by carrier gas molecules occurs and Boyle's law is obeyed. However, this does not invalidate the application of linear regression (which has been performed in order to simplify statistical analysis of data), since inspection of the graph of $y$ against $x$ for the relationship $y = {}^1/x$ shows that for large and small values of $x$ the curve is virtually linear. Investigation over a wider range of variation in atmospheric pressure (lower pressures in particular) should reveal the expected hyperbolic relationship. Exact interpretation of the situation is complicated by the way in which the thermal conductivity of the mixture of carrier gas and sample in the detector cavity varies with pressure.

The use of a technique simulating 'stopped-flow' injection (see the lowest plot in figure 9 and the regression data in table 2) gives rise to a considerably reduced effect of atmospheric pressure on peak area response. Thus, there is an increase of only 0·2% in response for an increase in pressure of 0·8% over a period of 60 days. This is good precision, even disregarding the known changes in atmospheric pressure, and is considered to be due to the thermal stability imparted on the contents of the sample loop by the column oven. However, the comments made previously in this paper concerning the residence time of the sample in the loop, would be relevant if the residence time were to vary. The somewhat poorer correlation between peak-area response and atmospheric pressure, which was obtained for injections made always at the prevailing atmospheric pressure, is considered to be due to the extra contribution to experimental error caused by resetting the digital barometer to the prevailing atmospheric pressure.

For a detector that is solely mass-sensitive, variations in atmospheric pressure should influence response mainly by altering the mass of sample injected [2]. A 'stopped-flow' technique of injection should then reveal significant changes in response as atmospheric pressure varies. The flame-ionization detector also suffers from the influence of atmospheric pressure on ionization efficiency [3], which may reinforce or oppose the effect at the sample loop, depending on the chosen chromatographic conditions and gases analysed. Thus, for a flame-ionization detector, compensatory effects may well change direction with increasing atmospheric pressure [3] so that for a 'stopped-flow' technique of injection the chromatographer sees a net effect which, over one range of atmospheric pressure, is due to the sum of two effects, and over another, to their difference.

Because of the incompressibility of liquids, variations in atmospheric pressure will not be able to influence the volume, and hence the mass, of liquid sample injected. Use of a thermal-conductivity detector when analysing liquid samples is therefore predicted to produce the same type of variation in peak-area response with changing atmospheric pressure as that shown in figure 9 for injections made at absolute pressures, independent of atmospheric pressure. Also, use of a solely mass-sensitive detector when analysing liquid samples should then produce no effect of atmospheric pressure variation at all, whilst use of a flame-ionization detector should reveal the effect that variation in atmospheric pressure has on response through its influence on ionization efficiency alone. It is true to say, however, that compensation for any changes in response which might occur will be possible through use of a technique involving internal standardization, and the poorer precision obtained when carrying out syringe injections should render less significant those changes in response that are due to variation in atmospheric pressure. Nevertheless, when analysing liquids it is worth knowing that there is predicted to be a contribution to variation in response from variation in atmospheric pressure.

When a digital barometer is employed for measuring injection pressure, a differential pressure transducer is preferred, since the operator then does not have to alter the Comparator set-point to be equal to the prevailing atmospheric pressure before each injection. It can simply be left at zero; this facilitates automation of the analysis. However, the resultant absolute pressure of gas contained in the sample loop will then be equal to the sum of the digital barometer setting and the prevailing atmospheric pressure, so that in order to inject a constant mass of gas, the Comparator set-point will have to be altered by an amount equal to the change in atmospheric pressure, whenever that pressure changes. The use of an absolute pressure transducer in this investigation has facilitated automation of injection of a constant mass of gas, and whilst the Comparator set-point *has* been altered to the prevailing atmospheric pressure in order to inject this pressure of sample gas, a 'high-purge', 'stopped-flow' technique of injection could have been used instead. It is also possible to switch from one transducer to the other.

It should be said, however, that it is only when injecting *at* the prevailing atmospheric pressure that the diluting effect of carrier gas molecules on the concentration of eluted sample in the detector cavity will compensate for the reinforcing effect due to the increased mass of sample in the sample-loop. When the digital barometer setting is adjusted to a value other than zero, for example $\pm$200 mbar, the proportional change in pressure in the sample loop, caused by changes in atmospheric pressure, does not equal the corresponding proportional change in pressure at the detector. For injections made at subatmospheric pressure, the reinforcing effect at the sample loop may then outweigh the diluting effect at the detector; for injections made at above atmospheric pressure the converse may apply.

The justification for considering the effect of variations in atmospheric pressure is shown by an analysis of atmospheric pressure data supplied by the London Weather Centre in Holborn. For the period of the investigation (61 days) this has revealed progressive drops in pressure of as much as 16 mbar (about 1·5%) in 39 h and progressive rises of 15 mbar in 63 h. Pressure/time gradients were as high as 3 mbar (0·3%) in 3 h and as low as $<$0·2 mbar (0·02%) in 12 h. Analysis of data covering a period of one year showed that atmospheric pressure varied from a minimum of 976 mbar to a maximum of 1036 mbar, a range of about 3%. This is not suggested to represent an exhaustive analysis of the variability of atmospheric pressure. The probability of a particular pressure, and rate of change of pressure, occuring, would be revealed only by analysing data obtained over a considerable period of time.

In view of the comments concerning the variability of atmospheric pressure, it can be seen that for most analyses where a 'stopped-flow' technique of injection is employed, the injection pressure will be the same as the detection pressure. However, for particularly long analyses, and depending on the rate of change of atmospheric pressure with time, these pressures could differ. Further, the relevant pressures could then differ for different components in the same chromatographic analysis. Thus, even though (for a thermal-conductivity detector) compensatory effects have been shown to minimize the effect of variations in atmospheric pressure on response, a long analysis carried out during turbulent weather could give rise to over- or under-compensation so that a net effect on response would be observed. The current work has not sufferred in this way because of the very short retention time of nitrogen. It is true to say, however, that where changes in atmospheric pressure do not occur during a period of time equal to about three times that for a complete analysis, the analysed sample can always be 'bracketed' by calibration samples. This eliminates the effects on results of longer-term variations in atmospheric pressure [5].

The work reported here shows that infrequent calibration could give rise to random errors in results, and that, depending on the technique of injection and type of detector employed, the use of absolute response factors may require measurement of atmospheric pressure.

### Conclusions

A specially constructed microcomputer-controlled sample-injection system is shown to be suitable for achieving any desired pressure of sample gas in the sample loop of a gas chromatograph, between vacuum and 25 lbf/in$^2$.

The microcomputer facilitates experimentation by providing a highly flexible way of controlling injection technique with special software. This software also aids optimization of column switching and back-flushing times in the associated automatic chromatographic anlaysis when combined with specially constructed hardware, and BASIC software in an SP4100 integrator.

Use of a combination of an absolute pressure transducer with a specially built digital barometer allows the influence of variations in atmospheric pressure on chromatographic response to be confined to the detector cavity. The results obtained show that there is a linear, negative correlation between peak-area response and atmospheric pressure for injections of nitrogen at absolute pressures of 1080, 1180, 1390 and 1690 mbar when using a thermal-conductivity detector with helium carrier gas. The linear, negative correlation is explained by the diluting action of carrier gas molecules on the concentration of sample in the detector cavity even though this would at first appear to demand a rectangular hyperbolic relationship between peak-area response and atmospheric pressure. This explanation is shown to be justified by the observed correlation—inspection of the curve for a rectangular hyperbola reveals that the curve is virtually linear at its extremities. The results could thus conceivably be at the upper extremity of each of the complete hyperbolic curves for the particular chromatographic system used in this investigation. Investigation over a wider range of variation in atmospheric pressure (lower pressures in particular)——might reveal the expected hyperbola.

The use of an absolute pressure transducer has not prevented an accompanying investigation into the effects of variation in atmospheric pressure on response when injecting *at* atmospheric pressure. When using the 'stopped-flow' injection technique the chromatographer can always measure the prevailing atmospheric pressure and then set the digital barometer to this value before injection. However, the use of a differential transducer would aid further automation of the 'stopped-flow' injection procedure but would then require resetting of the digital barometer to maintain a constant mass of gas in the sample loop.

Use of 'stopped-flow' injection is shown, when nitrogen is analysed with a thermal-conductivity detector, to give rise to only a very small change in peak-area response with varying atmospheric pressure. This is explained by the operation of two mutually compensatory effects of variation of atmospheric pressure on response, which act at the sample loop and detector simultaneously. The reinforcing effect on response through behaviour at the sample loop is compensated for by the diluting effect of carrier gas molecules at the detector.

However, where a component with a long retention time is analysed during periods of rapidly changing atmospheric pressure, the effects on response of changes in atmospheric pressure at the sample loop will be over-, or under-, compensated for by the corresponding changes in atmospheric pressure at the detector. Under such conditions, there could be significant changes in response for a thermal conductivity detector. Further, the atmospheric pressure at the detector could then be different for different components in the analysed mixture, with similar consequences.

It is predicted that when a solely mass-sensitive detector is employed, variations in atmospheric pressure will alter response mainly through an effect on the mass of sample injected. For a flame-ionization detector, variations in atmospheric pressure are predicted to influence response additionally through their effect on ionization efficiency, and the net effect on response will depend on the chromatographic conditions and gases analysed. The effects of variation in atmospheric pressure on chromatographic response can be eliminated by 'bracketing' the analysed sample with calibration samples, provided significant changes in atmospheric pressure do not occur during a period of time equal to about three times that for a complete analysis.

Predictions are also made about the effects of variation in atmospheric pressure on chromatographic response when liquids are analysed. In this case, the characteristic incompressibility of liquids renders them prone to a type of chromatographic behaviour which is similar to that observed when analysing gases using a thermal-conductivity detector with an absolute pressure transducer employed for maintaining a constant mass of sample in the sample loop. Again, compensation for any changes in response will be possible—for example by using a technique of internal standardization. Further, the poorer precision characterizing syringe injections renders such changes less significant.

An analysis of atmospheric pressure data from the London Weather Centre has revealed that during a 61-day investigation atmospheric pressure decreased by as much as 1·5% in 39 h, and increased by the same amount in 63 h. Pressure/time gradients were sometimes as high as 0·3% in 3 h, and as low as <0·2% in 12 h. Analysis of data covering a period of one year, showed that atmospheric pressure exhibited extremes of 976 and 1036 mbar which represents a range of approximately ± 3%. These figures show that changes in atmospheric pressure are worthy of consideration when carrying out chromatographic analyses.

The determination of the frequency of occurrence of particular changes in atmospheric pressure will necessitate analysis of data obtained over a considerable period of time; the analysis presented here is not intended to be an exhaustive one.

### Recommendations

Since modern integrators record the absolute time of injection on the chromatogram and on the report of the analysis, it is very likely that chromatographers throughout the world will have the necessary data to enable them to perform a check on correlation between chromatographic response and the ambient atmospheric pressure. They can record the pressure themselves or obtain it from a local weather station. The authors would be interested in communicating with anyone who might have such data, or who may have already made such checks on correlation.

### Acknowledgements

A shorter version of this paper was published in the March 1983 (Vol. 20, p. 102) issue of *Analytical Proceedings* (Royal Society of Chemistry, London).

The authors wish to thank the British Gas Corporation for permission to publish the present paper.

### References

1. Hooimeijer, J., Kwantes, A. and van de Craats, F., In the *Proceedings* of the second symposium organized by the Gas Chromatography Discussion Group under the auspices of the Hydrocarbon Research Group of the Institute of Petroleum and the Koninklijke Nederlandse Chemische Vereniging (Royal Tropical Institute, Amsterdam, 19–23 May 1958), p. 290.
2. Novák, J., Quantitative analysis by gas chromatography. *Chromatographic Science Series, Dekker*, **5** (1975), 18.
3. Boček, P., Novák, J. and Janák, J., *Journal of Chromatography*, **43** (1969), 431.
4. Deans, D. R., Huckle, M. T. and Peterson, R. M., *Chromatographia*, **4** (1971), 279.
5. Goedert, M. and Guiochon, G., *Journal of Chromatographic Science*, 7 (1969), 323.

### Appendix 1. Instrumentation and conditions

1.1 *Chromatographic*

| | | |
|---|---|---|
| Oven | Servomex | Type AO221 |
| Temperature controller | Servomex | Type TC201 |
| Solenoid valve programmer unit | Servomex | Type FS241 |
| Detector | Gow-Mac | Dual hot-wire |
| Detector power supply | Gow-Mac | Model 40–201 (constant voltage) |
| Gas sampling valve | Servomex | Six-port, type S.V. 233 |
| Integrator | Spectra Physics | Model SP4100 (with BASIC) |

1.2 *Electro-pneumatic Interface 2*

| | | |
|---|---|---|
| Solenoid valves 12 to 16 (24 V–1·3 W) | Festo Pneumatic | Solenoid head, type MKNC-022-3 Valve body, type LC-3-$\frac{1}{8}$ |
| Switch S1 | Festo Pneumatic | Pneumatic switch, type PEV-$\frac{1}{4}$ |
| Relay R1 | Radio Spares | 20A heavy duty, type 348-403 |

1.3 *Sample gas control valves*

| | | |
|---|---|---|
| Pneumatic valves 12 to 16 | Festo Pneumatic | Type VL/O-3-$\frac{1}{8}$, Special design SA 1392 for vacuum operation |
| Needle valves $NV_3$, $NV_4$ and $NV_5$ | Festo Pneumatic | Type GRO-$\frac{1}{8}$, 0-10 bar |
| Coarse control needle valve $NV_1$ | Hoke International | Type 1315 G2B |
| Fine control needle valve $NV_2$ | Hoke International | Type 1335 G2B (with digital handle) |

1.4 *Pressure measurement and control*

| | | |
|---|---|---|
| Transducer | Setra Systems from Lintronic | Model 204, range 0-25 psia, variable capacitance, excitation 24 V/output 5 V. (Intrinsically safe design recommended.) |
| Barometer | Lintronic | $3\frac{1}{2}$ Digit Display, reading in millibar, and providing auxiliary Parallel BCD Output and 6 Relay Contact closures |
| Converter | Lintronic | Taking parallel BCD output from barometer and providing: parallel BCD output; Serial 20 mA current loop output (110 or 300 Baud); Serial RS 232C output (110 or 300 Baud); and 5 V reference voltage for calibration of barometer |

1.5 *Electronic components* (all supplied by Radio Spares)

| *Component* | *Function location* | *Catologue number* |
|---|---|---|
| Integrated circuits: | | |
| IC10 | 7407 Hex buffer, open collector | 306–336 |
| OI10 and OI11 | Quad opto-isolators; on solenoid control board 5 | 307–064 |
| Relays: | | |
| RY7 to RY11 | Double pole DIL reed relays, form C; on solenoid control board 5 | 349–434 |
| Switches: | | |
| SW9 and SW10 | DIL, SPDT; in Interface 3, to select the source of hardware satisfying software interrupts | 337–554 |
| SW1 to SW4 | Manual interrupt overrides in Interface 1 | 316–989 |
| SW11 to SW15 | Manual overrides, controlling pneumatic valves in electro-pneumatic Interface 2 | 316–989 |
| RS1 | Remote start, push-button switch; on front panel of Interface 1 | 339–235 |
| Miscellaneous: | | |
| Two-tone alarm | In Interface 1; informs operator that certain stages have been reached in the analysis | 249–429 |
| Digital counter | In Interface 1; serves as a program step-number, or run-number indicator | 258–798 |
| Resistor networks | On solenoid control board 5, in Interface 2: | |
| | RN1 [150 Ω] | 140–013 |
| | RN2 [10 KΩ] | 140–057 |
| | RN3 [1 KΩ] | 140–035 |
| LEDs: | | |
| Amber | Solenoid control valve indicators, in Interface 2 | 587–709 |
| Red/green | Manual interrupt overrides, in Interface 1 | 586–728 |

1.6 *Chromatographic conditions*

| | |
|---|---|
| Primary column | Porapak T |
| Secondary column 1 | Molecular sieve 13X |
| Secondary column 2 | Porapak S |
| Oven temperature | 58°C |
| Carrier gas | Helium |
| Primary gas pressure | 27 psig (1·9 $Kg/cm^2$) |
| Secondary gas pressure | 20 psig (1·4 $Kg/cm^2$) |
| Sample loop volume | 1·3 $cm^3$ |

*Special conditions*

The normal elution order of carbon monoxide (after methane, on molecular sieve 13X), is reversed, by only partially activating the molecular sieve, by heating the packed column whilst carrier flows, at 175°C. for 4 h, after prior removal of fines in the bulk material by washing it in distilled water and then carrying out decantation.

### Appendix 2. Suppliers' addresses

2.1 *Chromatographic equipment*
Servomex Ltd, Crowborough, Sussex TN6 3DU, UK
Gow-Mac Instrument Company Ltd, Gillingham, Kent, UK

2.2 *Data handling*
Spectra Physics Ltd, 17 Brick Knoll Park, St. Albans, Hertfordshire, UK

2.3 *Pneumatic control*
Festo Pneumatic Ltd, Catherine Wheel Road, Brentford, Middlesex TW8 8BB, UK
Hoke International Ltd, 1-3 Bouverie Road, Harrow, Middlesex HA1 4HB, UK

2.4 *Pressure measurement and control*
Setra Systems Inc., 1 Strathmore Road, Natick, Massachusetts 01760, USA
Lintronic Ltd, 54-58 Bartholomew Close, London EC1A 7HB

2.5 *Microcomputer*
Zilog (UK) Ltd, Nicholson House, Maidenhead, Berkshire, UK

2.6 *Electronic components*
Radio Spares Ltd, 13-17 Epworth Street, London EC2P 2HA.

